# Specialty Grand Challenge for Brain Disease Mechanisms

**DOI:** 10.3389/fnmol.2021.689903

**Published:** 2021-05-10

**Authors:** Detlev Boison

**Affiliations:** Department of Neurosurgery, Robert Wood Johnson Medical School, Rutgers University, Piscataway, NJ, United States

**Keywords:** disease burden, disease mechanism, disease modification, network pharmacology, epigenetics, global approaches, neurological disorders, CNS disorders

## Global Burden of CNS Disorders

The global burden of CNS diseases is expected to increase dramatically within the next decades. A recent position paper in the *Annals of Neurology* estimates the annual cost of neurological diseases affecting nearly 100 million Americans to be in the range of 800 billion dollars [including $37B for epilepsy, $86B for traumatic brain injury (TBI), and $110B for stroke] and calls for the development of preventative and disease-modifying therapies (Gooch et al., 2017). Indeed, conventional therapies are largely symptomatic and do not influence the genesis or progression of the disease. A major gap and challenge for the development of novel disease modifying therapies is the transition from target-centric (symptomatic) approaches to globally-acting multi-modal approaches, which are able to restore complex network function in the brain. It is therefore time to develop innovative new concepts to understand better the interconnectedness of the CNS and to translate new knowledge into novel therapeutics. To achieve this goal Big Data approaches will play a major role in connecting findings from a multitude of different mechanisms and disciplines. The multidisciplinary and integrative scope of *Brain Disease Mechanisms* will help to bring different ideas from different disciplines under one common roof.

## New Challenges as a Consequence of Covid-19?

The outbreak of the novel coronavirus disease 2019 (COVID-19) caused by Severe Acute Respiratory Syndrome CoronaVirus-2 (SARS-CoV-2) may lead to new challenges and to a further increase in the global burden of CNS diseases. It has now become clear that SARS-CoV-2 can also attack the nervous system (Zubair et al., 2020), and it is estimated that at least 40% of COVID-19 patients develop neurological complications (Liotta et al., 2020), including encephalitis, increased risk of stroke, and injuries due to lack of oxygen (Fridman et al., 2020; Kantonen et al., 2020; Paterson et al., 2020). A wide range of injuries to the brain in turn, including stroke, traumatic brain injury, and brain infection, are known to constitute a primary cause for the development of acquired epilepsies including temporal lobe epilepsy (Klein et al., 2018). Because the latent period for the development of acquired epilepsies in humans is in the range of months to years it is still too early to assess whether COVID-19 might be linked to a future increase in epilepsy cases.

An additional unknown are the consequences of SARS-CoV-2 infections during pregnancy. A leading hypothesis for the etiology of neurodevelopmental disorders such as autism or schizophrenia suggests that maternal immune activation caused by viral infections during pregnancy might play a major causative role for the derailment of developmental processes critical for normal brain development (Canetta and Brown, 2012; Lombardo et al., 2018). Again, it is still too early to assess whether SARS-CoV-2 infections during pregnancy can be linked to the development of autism or schizophrenia.

The examples shown above illustrate that brain diseases and associated etiologies and mechanisms are a constantly evolving field, which may need concerted efforts to accelerate new research directions.

## New Frontiers in Disease Mechanisms

Why should we be interested in disease mechanisms? The ultimate goal, obviously, is that better understanding of disease mechanisms leads to better treatment options for persons affected by brain disorders and to the reduction of the global health burden. Four areas deserve increased attention during the next decade:

### Disease Modification

The biggest challenge for the reduction of the global health burden of neurological conditions is a paradigm shift from symptomatic to disease modifying treatments. Up until now, pharmacological treatment options are largely symptomatic. For example, antiseizure drugs (ASDs) have been designed to reduce neuronal excitability, and thereby seizures, the dominant symptom of epilepsy. They do so mostly by affecting ion channels and neurotransmitter release (Sills and Rogawski, 2020). However, despite the development of about 30 new ASDs over the past 30 years, treatment outcomes for persons with epilepsy have not significantly been improved and about one third of all persons with epilepsy remain refractory to pharmacological treatment (Chen et al., 2018). Most currently used ASDs have been discovered in screens designed to detect seizure suppression in rodent seizure models. As a default of this screening approach, compounds are identified, which have the capability to suppress the dominant symptom of epilepsy, which is the seizure. As becomes obvious, seizure-based drug screens are not able to identify compounds, which can treat comorbidities of epilepsy, such as depression, anxiety, or cognitive impairment, or which affect disease progression and epilepsy development. Therefore, there is a major unmet need to identify treatment options, which are disease modifying and thereby affect fundamental mechanisms implicated in pathogenic processes leading to disease and its progression. Treatments, which prevent disease or its progression would be a game changer not only for epilepsy but also for progressive neurodegenerative diseases such as Alzheimer's or Parkinson's disease. In order to develop novel disease modifying treatments, a better understanding of the interconnectedness of the CNS on a multi-omics level becomes a necessity.

### Network Approaches

As opposed to the design of highly selective ligands that bind on individual targets, network pharmacology holds the promise to affect several beneficial targets simultaneously, an approach suitable to increase efficacy and reduce toxicity (Hopkins, 2008). Those network based approaches can be based on a rational design. If several mechanisms are involved in disease development and progression, it can be assumed that those mechanisms are connected by multiple nodes. Identification of central nodes and targeting them in a rationally designed network approach holds the promise to reconstruct network function implicated in disease pathogenesis. A proof of principle for the utility of this approach has been demonstrated recently by demonstrating that a combination of levetiracetam and topiramate altered multiple epileptogenesis-relevant targets and provided robust disease modifying effects (Schidlitzki et al., 2020). A promising alternative to target-centric conventional pharmacology is the development of biochemical interventions for the treatment of disease. It is now well-established that biochemical alterations are implicated in the pathophysiology of a majority of neurological conditions (Boison, 2016). Specifically, core metabolites, such as adenosine are uniquely linked to energy homeostasis (ATP), used as building blocks of biomolecules (RNA, including poly-A tails of mRNAs), coupled to transmethylation reactions (DNA and histone methylation), and act as receptor ligands (adenosine receptors) (Boison, 2013; Boison and Yegutkin, 2019). Thus, adenosine is a unique network regulator linking metabolism and gene expression with neuromodulation. Strikingly, adenosine deficiency is a common pathological hallmark of epilepsy, Alzheimer's disease, Parkinson's disease, amyotrophic lateral sclerosis, and can be the direct explanation for a wide range of symptoms including seizures, sleep alterations, depression, and changes in cognition and affective behavior (Li et al., 2008; Shen et al., 2012; Hines et al., 2013; Boison and Aronica, 2015). Consequently, adenosine augmentation therapies, e.g., through engineered stem cells (Fedele et al., 2004; Li et al., 2009), are uniquely suited to restore network homeostasis, and to not only suppress comorbid symptoms but also to exert lasting disease modifying therapeutic effects (Li et al., 2008; Boison, 2012; Shen et al., 2012; Williams-Karnesky et al., 2013). The concept of network approaches is not new. This year marks the 100th anniversary of the high-fat, low-carbohydrate ketogenic diet, a metabolic therapy, which has successfully been used for the treatment of epilepsy for decades (Neal et al., 2008; Kossoff, 2010; Stafstrom and Rho, 2012). Recent studies suggest strongly that metabolic approaches may have underappreciated antiepileptogenic and disease-modifying properties (Muller-Schwarze et al., 1999; Todorova et al., 2000; Lusardi et al., 2015; Boison, 2017; Boison and Rho, 2020) and provide benefits to a wide spectrum of additional conditions including pain, autism, brain cancer, and Alzheimer's disease (Ruskin et al., 2009, 2017; Brownlow et al., 2013; Chung and Park YRationale, 2017; Vergati et al., 2017). The benefits of metabolic therapies can be explained by combining several different mechanisms which affect network homeostasis on the levels of energy equilibrium, mitochondrial function, changes in metabolites and neurotransmitters, and epigenetic reprogramming (Kobow et al., 2013; Rogawski et al., 2016; Boison, 2017; Augustin et al., 2018; Boison and Rho, 2020). The examples above demonstrate the promise of network based treatment approaches and suggest that the biochemistry of brain disease is a new frontier to understand the complexity and interconnectedness involved in etiopathological processes. Fully understanding those mechanisms will provide the basis for rationally designed multimodal therapeutics uniquely suited to exert disease modifying properties and to treat complex comorbid syndromes.

### Epigenetics

We are living in the post genomic age. Genetic mutations and polymorphisms yield cues for our understanding of CNS disorders and the development of personalized medicines. A recent PubMed search for “genomic” yielded 1.6 million hits. In contrast, the term “epigenomic” yielded only 18,000 hits. This discrepancy is surprising, given the fact that epigenetic alterations, which include modifiable changes in DNA methylation, histone methylation and acetylation, and the expression of non-coding RNAs, regulate the expression of the very genes that are the focus of genomics-based research efforts. Because epigenetic mechanisms regulate gene expression, there is an urgent need to invest in the field of epigenomics which is one of the remaining frontiers in neuroscience. The study of epigenetics and the development of “epigenetic medicines” is relatively well-developed in the field of cancer (Du et al., 2015; Huang et al., 2015; Zahnow et al., 2016), however in its infancy in our understanding of neurological disorders. For the rigorous assessment of epigenomic data sets it will be important to standardize data and methods as even minor protocol variations can have major impact on epigenomic data sets. Epigenetic changes are subject to metabolic regulation (Kobow et al., 2013; Williams-Karnesky et al., 2013; Boison and Rho, 2020; Kuchukulla and Boison, 2020) and thus may provide an interface between environment, metabolism, and gene expression. Of crucial importance for the understanding of disease mechanisms is the interconnectedness of physiological, molecular, cellular, metabolic, epigenetic, and genetic mechanisms at the subcellular, cellular, and regional levels.

### Global Approaches

Finally, the burden of CNS diseases is global with tremendous regional disparities. It is not enough to focus on Alzheimer's disease, which is a prevalent problem specifically in wealthy countries with a high and rising life expectancy. Strikingly, according to data from the World Health Organization, Alzheimer's disease is five times more prevalent in high income countries as compared to low income countries, whereas meningitis is 13 times more prevalent in low income countries as compared to high income countries. Other, neglected conditions, which affect a significant share of the world population require equal attention. For example, cerebral malaria, the most severe neurological manifestation of severe malaria, has an incidence of 1,120/100,000/year in endemic areas of Africa. It is estimated that a minimum of 575,000 children in Africa develop cerebral malaria annually (Breman, 2001; Murphy and Breman, 2001). Consequences of cerebral malaria include long-term cognitive impairment (25%), speech and language impairment (11.8%), epilepsy (10%), as well as behavioral and neuropsychiatric disorders (Idro et al., 2010a,b). Those examples show major disparities in the distribution of the global burden of neurological diseases; therefore it becomes a moral necessity to invest more research into global health issues. Related to this, there is a risk of introducing bias in clinical trial design. It is important to design trials appropriately to make sure that resulting treatments work equally well across diverse population groups.

## The Need for Public Engagement

The current COVID crisis has shown that public distrust in science can have catastrophic impacts on case numbers. The combination of distrust in science and medical populism has led to avoidable surges in COVID infections and deaths, specifically in countries where leaders have discredited scientific knowledge and findings, by downplaying the impacts of the pandemic, by promoting easy and scientifically unfounded solutions or treatments, and by forging divisions between believers and non-believers of science (Lasco, 2020; Hotez, 2021). On the other hand, science has produced a remarkable success story: the development of new vaccines to a new virus within a record breaking time frame of <1 year. There is hope that, if the vaccines work in significantly reducing the impacts of the pandemic and allow the return to a new normal, there will be a boost for the general trust in science. Currently, about three quarters of the population agree that science and technology make our lives better and that scientists contribute to major medical advances. This implies that scientists have a responsibility to maintain and expand this level of trust by serving the public. It means that the fruits of scientific discovery need to be shared broadly with our communities and that scientists need to build trust by demonstrating that science is not done in silos, but that scientists are members of the public and that the public is part of the scientific community. Engaging the public will be key in fighting the pandemic and there is hope that advances in fighting the pandemic will instill trust in the scientific process.

## Conclusions

*Frontiers in Molecular Neuroscience* is a leading journal in the area of Neuroscience publishing rigorously peer-reviewed articles with a focus on molecular mechanisms implicated in health and disease of the nervous system. Its *Brain Disease Mechanisms* section focuses on key pathways and molecular mechanisms involved in the genesis, progression, and maintenance of central nervous system pathologies including neurodevelopmental, neurodegenerative, neuroinflammatory, and neuropsychiatric diseases, including insults such as stroke, traumatic and spinal cord injuries, and infection, as well as mechanisms linking injury to downstream consequences such as epilepsy or neuropathic pain. Of crucial importance for the understanding of disease mechanisms is the interconnectedness of physiological, molecular, cellular, metabolic, epigenetic, and genetic mechanisms at the subcellular, cellular, and regional levels. A specific interest is the exploration of disease mechanisms and their translation into novel targeted therapeutic approaches. *Brain Disease Mechanisms* thereby provides an interdisciplinary platform for new developments in this highly complex field that demands the involvement of a broad range of professionals and the public to create a forum for the exchange of knowledge and the global dissemination of science.

## Author Contributions

DB designed and wrote the manuscript.

## Conflict of Interest

The author declares that the research was conducted in the absence of any commercial or financial relationships that could be construed as a potential conflict of interest.
